# Multidisciplinary Management of a Permanent Maxillary Lateral Incisor Fused to a Palatally‐Positioned Supernumerary Tooth With Shared Pulp Anatomy: A Case Report

**DOI:** 10.1002/ccr3.73379

**Published:** 2026-08-19

**Authors:** Mehrfam Khoshkhounejad, Maryam Babaahmadi, Somayeh Majidi

**Affiliations:** ^1^ Department of Endodontics, School of Dentistry Tehran University of Medical Sciences Tehran Iran; ^2^ Dental Research Center, Dentistry Research Institute Tehran University of Medical Sciences Tehran Iran; ^3^ Faculty of Dentistry of Tehran University of Medical Sciences Tehran Iran

**Keywords:** cone‐beam computed tomography, multidisciplinary treatment, supernumerary tooth, tooth fusion

## Abstract

Fusion between a permanent tooth and a supernumerary tooth is a rare developmental anomaly, particularly when the teeth share pulp anatomy, making diagnosis and treatment challenging. This case involved a 10‐year‐old boy with an esthetic concern caused by a supernumerary tooth fused to the palatal aspect of the permanent maxillary left lateral incisor. CBCT imaging revealed a rare configuration consisting of a shared coronal pulp chamber with separate apical constrictions. A multidisciplinary, phased treatment approach was implemented, including endodontic therapy with placement of an MTA apical barrier, surgical recontouring while preserving periodontal tissues, and orthodontic space redistribution. At the 12‐month follow‐up, clinical and radiographic evaluation showed favorable healing, stable periodontal and periapical health, satisfactory esthetics, and high patient and parent satisfaction, as assessed by informal verbal feedback and clinical interview at follow‐up visits. This case highlights that careful multidisciplinary planning and staged endodontic, surgical, and orthodontic management can successfully treat complex fusion anomalies, preserving function, esthetics, and periodontal health.

## Key Clinical Message

Fusion of a permanent lateral incisor with a palatally‐positioned supernumerary tooth and shared pulp anatomy requires a phased multidisciplinary approach—endodontics, surgery, and orthodontics. CBCT is indispensable for precise diagnosis and treatment planning. Coordinated care successfully restores function, periodontal health, and smile esthetics.

## 1 Introduction

Developmental dental anomalies can present significant diagnostic and therapeutic challenges, particularly when multiple anomalies coexist within a single tooth. Among these, supernumerary teeth are relatively uncommon, occurring in 0.1%–3.8% of the permanent dentition, with a marked predilection for the anterior maxilla [1]. Tooth fusion, defined as the union of two normally separate tooth germs during odontogenesis, is another rare developmental anomaly, with a reported prevalence of 0.2%–2.5%, occurring more frequently in the primary dentition than in the permanent dentition [1]. Fusion between a permanent tooth and a supernumerary tooth is considerably rarer, with an estimated prevalence of approximately 0.1%, and most commonly involves the maxillary anterior region [2].

Although the exact etiology of tooth fusion remains uncertain, several mechanisms have been proposed, including physical pressure between adjacent developing tooth germs, persistence of the interdental lamina, genetic predisposition, and developmental trauma [3, 4, 5, 6]. Depending on the stage of odontogenesis at which union occurs, fusion may be complete or incomplete, resulting in a wide spectrum of crown and root morphologies (Table 1) [1, 7].

**TABLE 1:** Morphological classification of tooth fusion.

Type	Crown morphology	Root morphology
Type 1	Bifid crown	Single root
Type 2	Enlarged crown	Enlarged root
Type 3	Two fused crowns	Double conical root
Type 4	Two fused crowns	Two fused roots

The clinical presentation of a permanent tooth fused with a supernumerary tooth varies according to its morphology and location but frequently includes an enlarged or malformed crown, malocclusion, impaired oral hygiene, and an increased risk of caries and periodontal complications. These anomalies may also compromise esthetics, negatively affecting patients' self‐confidence and quality of life, particularly when the anterior dentition is involved [8]. Furthermore, their complex anatomy may complicate endodontic, restorative, orthodontic, and surgical treatment, making accurate diagnosis and individualized multidisciplinary treatment planning essential [9].

This report describes the multidisciplinary management of a permanent maxillary lateral incisor fused to a palatally positioned supernumerary tooth, an uncommon developmental anomaly with significant diagnostic and therapeutic implications. Notably, the patient's primary reason for seeking treatment was dissatisfaction with the esthetic appearance of the affected tooth rather than functional symptoms, highlighting the considerable cosmetic and psychosocial impact that such anomalies may have. This case emphasizes the importance of comprehensive diagnosis, careful treatment planning, and an interdisciplinary approach to achieving favorable esthetic and functional outcomes.

## 2 Case History /Examination

A 10‐year‐old male patient was referred to the Department of Orthodontics for evaluation of an esthetic concern localized to the anterior maxilla. His systemic medical history was noncontributory, and he reported no antecedent episodes of dental trauma or restorative treatment in the affected quadrant.

Intraoral examination revealed supragingival biofilm accumulation and multiple active enamel‐dentin carious lesions. Focused assessment of the maxillary left quadrant disclosed a supernumerary element closely approximated to the palatal aspect of the permanent lateral incisor, with clinical features suggestive of fusion. The fused dental complex exhibited physiological immobility (Miller's Class 0), probing pocket depths within normal limits (2–3 mm), and a vital pulpal response to both thermal (cold) and electric pulp testing. Percussion and digital palpation yielded negative findings, ruling out apical periodontitis or periapical pathology (Figure 1A,B). Radiographic evaluation, comprising panoramic view, confirmed the presence of a supernumerary element fused to the palatal aspect of the maxillary left permanent lateral incisor (Figure 1C). Both dental units exhibited complete root formation with closed apices, as evidenced by radiographic apexification.

**FIGURE 1:**
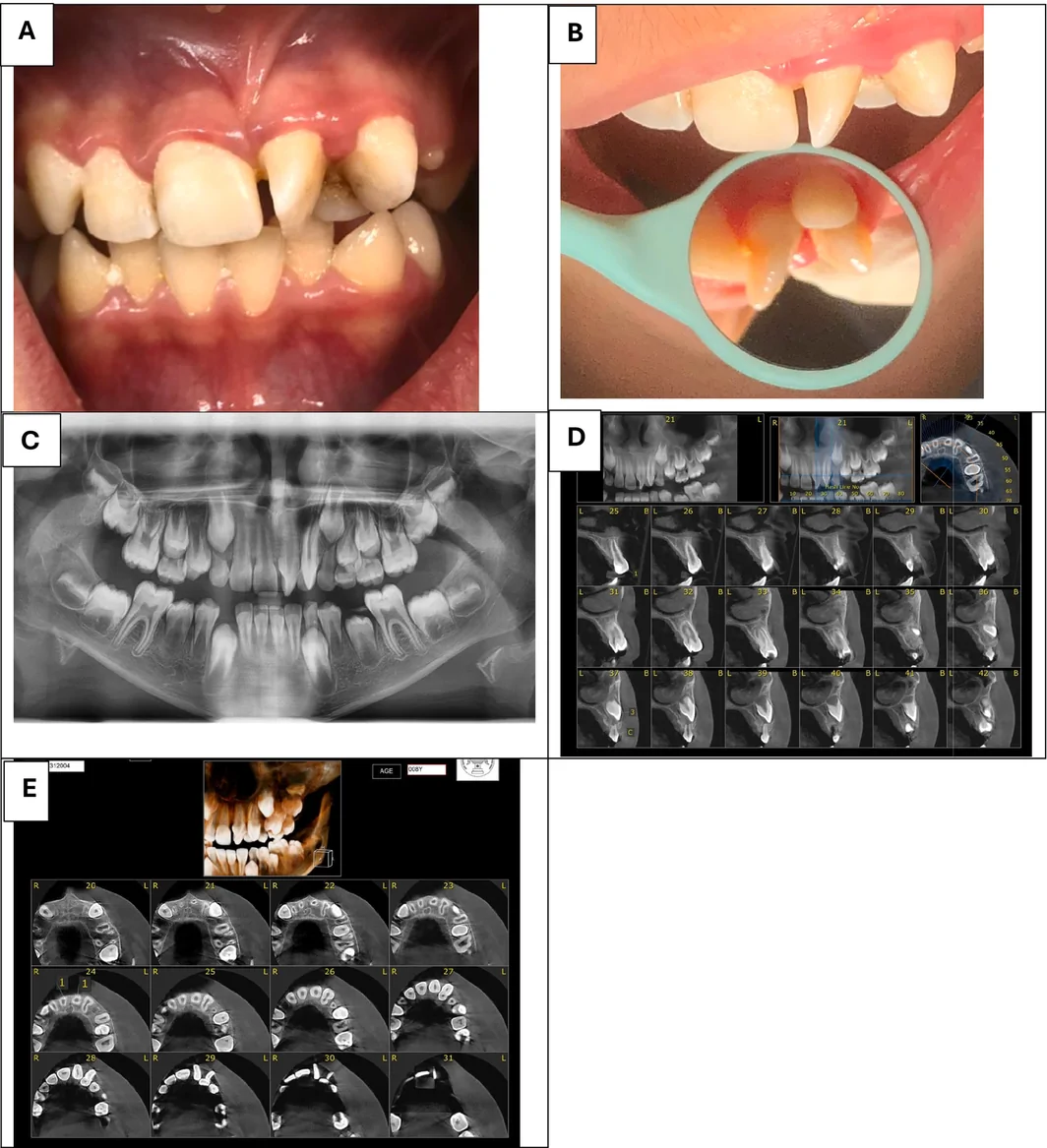
Preoperative clinical and radiographic documentation of the fused supernumerary‐permanent complex. (A) Frontal intraoral photograph demonstrating supragingival biofilm accumulation and generalized poor oral hygiene. (B) Occlusal view of the maxillary arch revealing the supernumerary element fused to the palatal aspect of the permanent left lateral incisor (tooth #22). (C) Panoramic radiograph confirming the presence of the fused supernumerary element palatal to the maxillary left lateral incisor, with complete root formation and closed apices in both dental units. (D) CBCT sagittal section demonstrating the shared coronal pulp chamber between the lateral incisor and the supernumerary element. (E) CBCT axial view illustrating the palatal position of the supernumerary element relative to the lateral incisor.

To precisely delineate the intricate root morphology and assess the pulpal topography, cone‐beam computed tomography (CBCT) was performed using a standardized protocol (voxel size: 0.2 mm; field of view: 5 × 5 cm). Multiplanar reformatting and three‐dimensional reconstruction revealed a distinct pulpal interconnection between the fused structures, characterized by a shared coronal pulp chamber with separate apical constrictions (Figure 1D,E). This anatomical configuration was deemed critical for treatment planning, as it necessitated a unified endodontic approach prior to any surgical intervention.

## 3 Diagnosis

Based on the combined clinical and radiographic findings, a definitive diagnosis of fusion between the permanent maxillary left lateral incisor (tooth #22) and a palatally‐positioned supplemental tooth was established. The differential diagnosis included gemination of tooth #22; however, the presence of a supernumerary element with a distinct periodontal ligament space and separate apical constrictions on CBCT conclusively favored fusion.

## 4 Treatment

The treatment was executed in a sequential, phased manner, commencing with endodontic therapy, followed by surgical recontouring, and concluding with comprehensive orthodontic intervention. Root canal treatment of the maxillary left lateral incisor (tooth #22) and the fused supernumerary element was performed under a dental operating microscope (Carl Zeiss, Germany) to ensure optimal visualization of the complex pulpal anatomy. Given the patient's age (10 years) and the presence of wide apical foramina in both dental units, a customized obturation technique was employed. The apical third of each canal was obturated with EdgeMTA cement (EDGE MTA, Hamerz, Iran) to establish an apical barrier and promote apical closure, while the remaining coronal and middle canal spaces were filled using thermoplasticized gutta‐percha (System B/Obtura II) with AH 26 epoxy‐resin sealer. Postobturation periapical radiographs confirmed satisfactory canal filling with no evidence of overextension or voids, and coronal access cavities were subsequently restored with composite resin (Figure 2A–F). Following endodontic therapy and a 4‐week healing period, periodontal surgery was performed to remove the supernumerary element and recontour the fused complex. Local anesthesia was achieved via an infraorbital nerve block using 2% lidocaine with 1:100,000 epinephrine. A sulcular incision was made, and a full‐thickness mucoperiosteal flap was elevated and reflected from the mesial aspect of the maxillary left first premolar to the mesial of the left central incisor, providing adequate surgical access to the palatal aspect of the fused complex (Figure 3A). The fusion groove was deepened using a needle bur, and the supernumerary crown was subsequently sectioned and separated in one piece using a fissure bur, with care taken to preserve the underlying periodontal attachment of the lateral incisor (Figure 3B–D). The exposed residual coronal pulp chamber was sealed with light‐cured glass ionomer cement (Fuji II LC Improved; GC Corporation, Tokyo, Japan) to prevent bacterial microleakage and protect the endodontic obturation (Figure 3E). The flap was then repositioned and secured with interrupted sutures, which were removed after 10 days to allow for adequate soft tissue healing (Figure 3F). At the 12‐month follow‐up (following the surgical procedure), clinical and radiographic evaluation revealed healthy periradicular tissues, normal probing depths (2–3 mm), and complete soft tissue healing with satisfactory gingival contour, with no evidence of recurrence or pathology.

**FIGURE 2:**
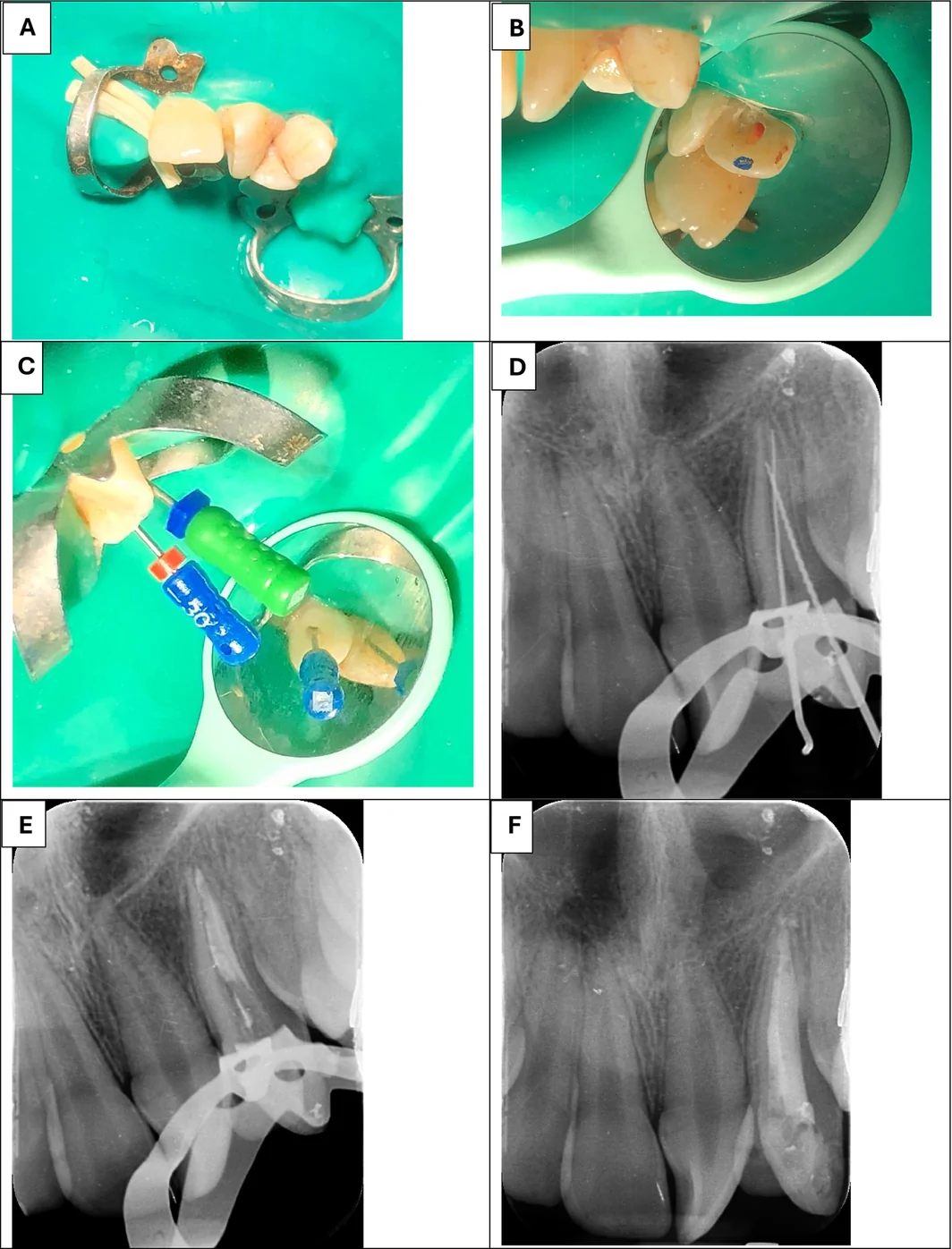
Endodontic management of the fused supernumerary‐permanent complex. (A) Preoperative clinical photograph of the maxillary anterior region prior to treatment initiation. (B) Intraoperative photograph showing the endodontic access cavity preparation, providing adequate visualization of the shared coronal pulp chamber. (C) Clinical photograph depicting the determination of working length using an electronic apex locator. (D) Periapical radiograph confirming the working length with a file positioned at the apical constriction of each canal. (E) Postoperative radiograph demonstrating the placement of the MTA apical plug extending to the mid‐root level, establishing an apical barrier. (F) Final periapical radiograph following obturation of the remaining canal space with thermoplasticized gutta‐percha and epoxy‐resin sealer, confirming satisfactory canal filling with no evidence of voids or overextension.

**FIGURE 3:**
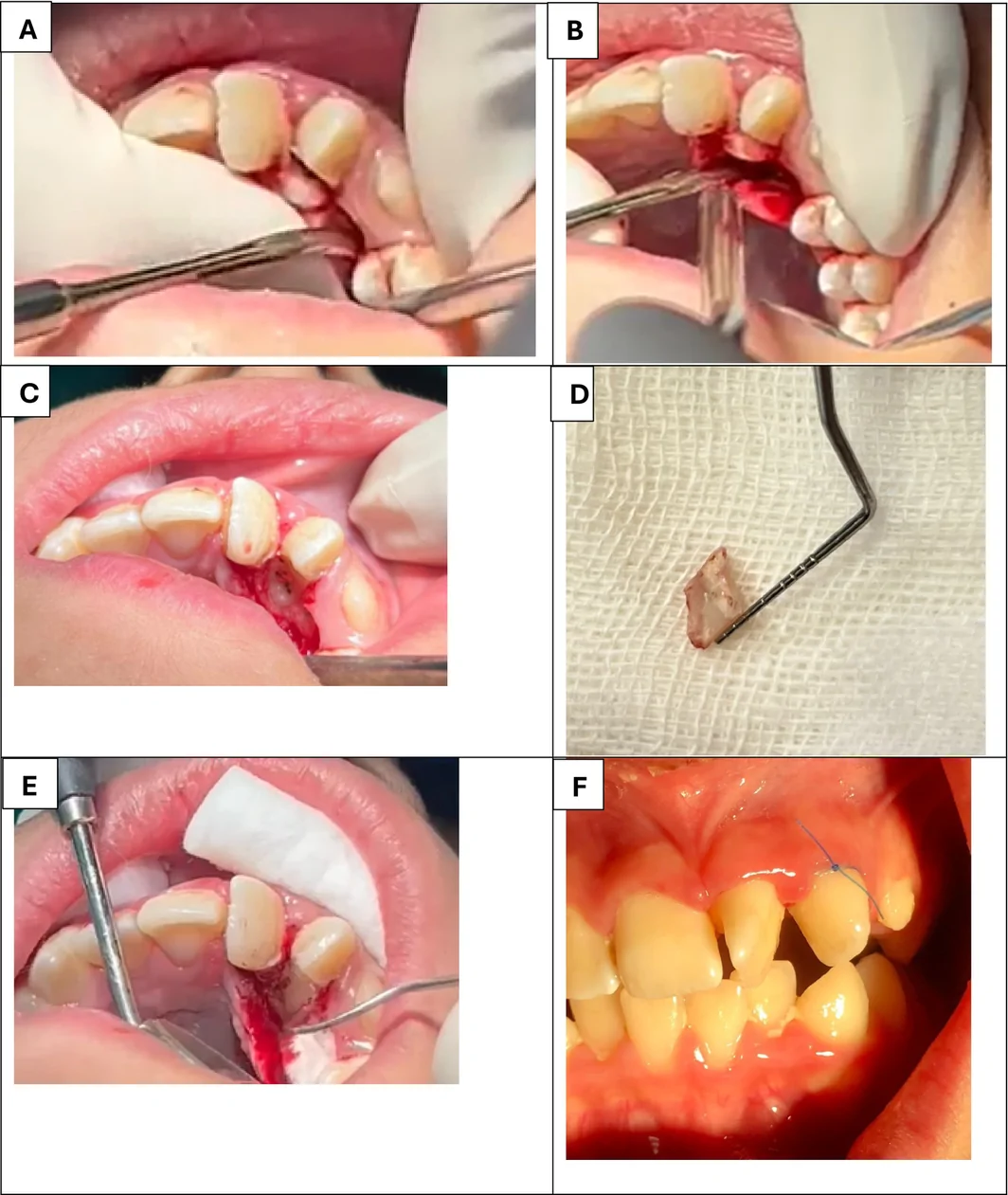
Surgical management of the fused supernumerary‐permanent complex. (A) Intraoperative photograph demonstrating the sulcular incision design along the gingival margin, extending from the mesial aspect of the maxillary left first premolar to the mesial of the left central incisor. (B) Full‐thickness mucoperiosteal flap elevation and reflection, providing adequate surgical access to the palatal aspect of the fused complex. (C) Intraoperative view showing sectioning and removal of the fused palatal supernumerary crown following deepening of the fusion groove with a needle bur. (D) The extracted supernumerary crown fragment, exhibiting an irregular morphology consistent with a supplemental tooth structure. (E) Application of light‐cured glass ionomer cement to seal the exposed residual coronal pulp chamber. (F) Clinical photograph at 10‐day postoperative follow‐up, demonstrating satisfactory soft tissue healing with complete flap adaptation and no evidence of inflammation or dehiscence.

Following complete soft tissue healing, comprehensive orthodontic therapy was initiated using fixed orthodontic appliances to address the residual esthetic and functional concerns. The initial phase of active orthodontic treatment involved the sequential creation of mesiodistal space through the controlled application of nickel‐titanium open‐coil springs and elastomeric chain mechanics, thereby facilitating the gradual repositioning of the maxillary left lateral incisor into its proper arch alignment (Figure 4). Subsequent orthodontic mechanotherapy is progressing with the objective of achieving complete arch coordination, optimal overbite and overjet relationships, and final esthetic integration of the treated dental unit within the maxillary arch. Interim clinical evaluation demonstrates satisfactory space redistribution and progressive alignment of the maxillary anterior segment, with the patient maintaining regular follow‐up visits for continued orthodontic supervision.

**FIGURE 4:**
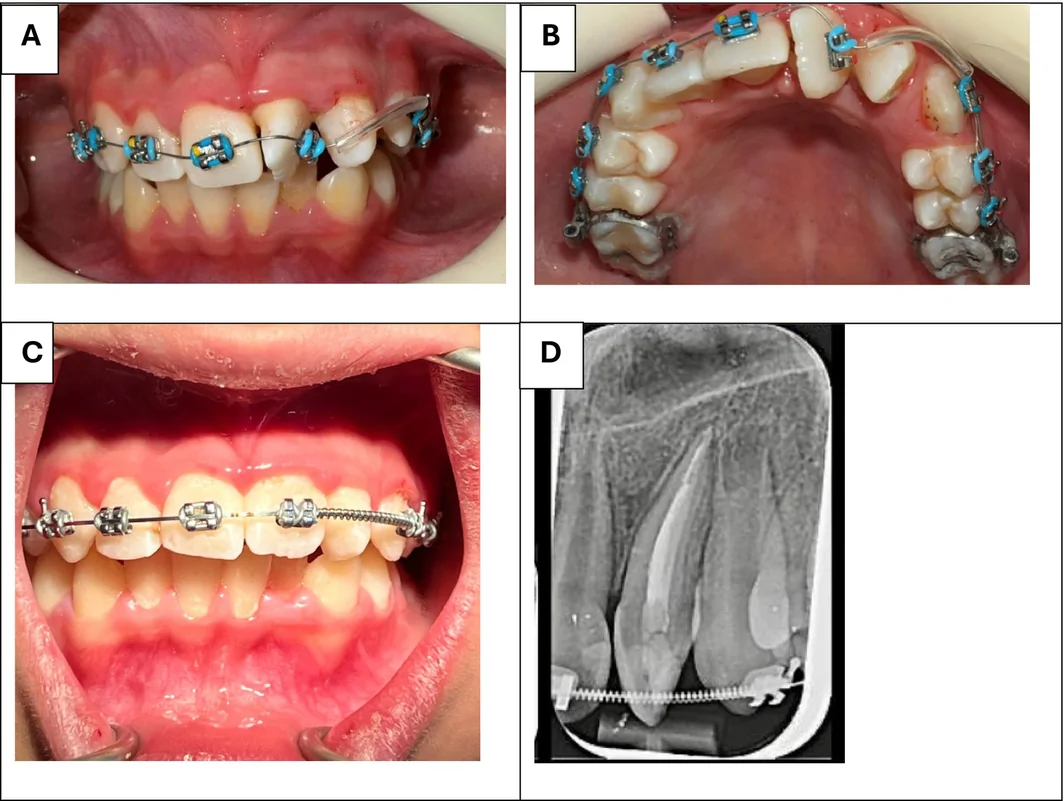
Interim orthodontic phase demonstrating progressive alignment of the maxillary anterior segment. (A) & (B) Intraoral photograph showing the initial stage of orthodontic mechanotherapy, with fixed orthodontic appliances in place and nickel‐titanium‐coil springs applied to create mesiodistal space, thereby facilitating the gradual repositioning of the maxillary left lateral incisor into proper arch alignment. (C) Subsequent intraoral photograph demonstrating progressive alignment of the maxillary central incisors following active orthodontic mechanotherapy, with satisfactory space redistribution and continued repositioning of the lateral incisor. (D) Follow‐up radiograph confirming intact lamina dura, with no periapical pathology or crestal bone loss.

Ultimately, the resolution of this patient's esthetic concern was achieved not through a single intervention but through the harmonious integration of endodontic, surgical, and orthodontic therapies, demonstrating that even complex anterior dental anomalies can be predictably managed to restore both function and smile esthetics when a coordinated, phased approach is employed.

## 5 Conclusion

This case demonstrates that a meticulously sequenced, multidisciplinary approach—integrating endodontic stabilization, surgical periodontal preservation, and orthodontic rehabilitation—can successfully resolve complex anterior dental anomalies involving fusion with a supernumerary element and shared pulp anatomy. The favorable clinical and esthetic outcomes underscore the value of team‐based care in managing such rare presentations, though long‐term follow‐up beyond the initial 12‐month postsurgical assessment remains essential to ensure sustained pulpal and periodontal health into adulthood.

## 6 Discussion

Fusion is a rare developmental dental anomaly characterized by the union of two separate tooth germs during odontogenesis. Although fusion may involve primary, permanent, or supernumerary teeth, the fusion of a permanent tooth with a supernumerary element is particularly uncommon, with a reported incidence of approximately 0.1%, predominantly affecting the maxillary anterior region [10]. Distinguishing fusion from gemination is diagnostically challenging. Accurate differentiation is essential, as fusion with a supernumerary tooth typically requires a multidisciplinary approach, whereas gemination may be managed conservatively.

The CBCT findings in this case—a shared coronal pulp chamber with separate apical constrictions—represent a rare anatomical variant that does not fit neatly into existing classification systems for root fusion. Zhang et al. [11], in their CBCT‐based study of maxillary molar morphology in a Chinese subpopulation, categorized root fusions into six distinct types: fusion of two roots (Types 1–3: MB‐DB, MB‐P, DB‐P), fusion of three roots (Types 4–6: MB‐DB‐P, DB‐MB‐P, MB‐P‐DB in a V‐shape), and a category for all roots fused in a Y or cone shape. However, this classification system was specifically designed for multirooted posterior teeth and does not adequately capture the complexity of coronal fusion between anterior teeth with shared pulp chambers. The present case more closely resembles a “complete fusion” as described by Cunha et al. [12], wherein two tooth germs unite early in odontogenesis, resulting in a shared coronal pulp space but maintaining separate apical exits. This distinction is clinically significant for multiple reasons. First, a shared coronal pulp chamber necessitates a unified endodontic approach—treating both units as a single pulp system—whereas separate pulp chambers might permit selective endodontic intervention. Second, the identification of separate apical constrictions on CBCT is particularly valuable, as it informs the decision to place MTA barriers in each canal independently while still addressing the coronal pulp as a continuous system. Furthermore, the developmental timing of fusion has a direct impact on treatment planning. When fusion occurs at an early stage, the root canal systems of the two teeth merge, resulting in a complicated morphology that may require more extensive endodontic treatment. Conversely, fusion occurring in the final stages results in two separate root canal systems, potentially allowing more conservative management [13]. In this case, the shared coronal pulp with separate apical constrictions suggests that fusion occurred at an intermediate stage of development, with early coronal union but relatively late root separation—a configuration that demands both a unified coronal approach and individualized apical management.

The clinical management of fused teeth, particularly those involving supernumerary components, is inherently complex and demands an individualized, interdisciplinary strategy. Documented treatment modalities range from conservative observation to orthodontic therapy, surgical intervention (sectioning or extraction), endodontic treatment, and restorative procedures [1, 8, 14].

Treatment planning depends on multiple factors, including the extent of fusion, root and pulp anatomy, esthetic demands, patient age, and overall dental health. The decision to perform prophylactic root canal treatment prior to surgical separation warrants explicit justification, given the tooth's positive pulp sensibility and absence of apical pathology. The primary rationale was anatomical: the presence of a shared coronal pulp chamber with thin dentinal walls created an unavoidable communication between the two units. Surgical sectioning of the supernumerary crown, as planned, would inevitably expose and breach the shared pulp chamber, leading to pulp devitalization of the retained lateral incisor fragment. Similar cases in the literature have reported that surgical division of fused teeth with a single shared pulp canal is contraindicated unless endodontic treatment is performed first, as sectioning carries a high risk of pulp exposure, perforation, and subsequent devitalization of the retained tooth [12]. Furthermore, the patient's age (10 years) and the presence of wide apical foramina necessitated a controlled endodontic protocol with apical barrier placement to achieve predictable sealing of the open apices. Performing endodontic therapy before surgical separation allowed the MTA to serve as both an apical barrier and a protective seal against bacterial contamination during the surgical exposure, reducing the risk of postoperative apical periodontitis [15]. This sequencing also ensured that any potential procedural complications (e.g., canal transportation, instrument fracture) could be managed before the surgical phase, thereby minimizing cumulative treatment risks. In the present case, the presence of a shared coronal pulp chamber with root canals converging apically—a rare anatomical configuration—necessitated a unified endodontic approach prior to surgical intervention, underscoring the critical role of precise anatomical characterization in treatment planning.

A distinctive feature of this case is the palatal presentation of the fused supernumerary element—less commonly reported than proximal or labial unions. This palatal position posed unique surgical access challenges and further complicated the orthodontic repositioning of the lateral incisor. CBCT proved indispensable in navigating these complexities, providing three‐dimensional visualization that was critical for assessing the level of union, root morphology, and pulpal topography, thereby guiding the sequential endodontic, surgical, and orthodontic treatment plan [16, 17]. The integration of CBCT into the diagnostic workflow not only facilitated accurate treatment planning but also minimized the risk of iatrogenic complications, including periodontal damage.

A review of comparable cases underscores the diversity of management strategies employed for fused supernumerary‐permanent complexes. Reported approaches range from conservative monitoring in asymptomatic cases to combined endodontic‐surgical‐restorative‐orthodontic interventions, particularly when esthetic compromise, pulpal involvement, or significant displacement is present [8, 12, 18]. However, few cases in the literature describe the successful application of a phased multidisciplinary approach—endodontic therapy with MTA apical barrier placement, followed by surgical recontouring with periodontal preservation, and culminating in orthodontic space redistribution—in a growing patient with thin dentinal walls, wide apical foramina, and a shared pulp chamber. This case, therefore, contributes a novel treatment algorithm that addresses both the biological and esthetic dimensions of such anomalies. Surgical separation of fused teeth carries inherent risks that must be carefully considered. The most significant complication is periodontal attachment loss, as the surgical division inevitably creates a denuded root surface adjacent to the extraction site. Olsen et al. [19] reported that surgical separation of supernumerary teeth fused to permanent incisor teeth has typically resulted in residual postsurgical periodontal defects, including loss of attachment and deep periodontal pocketing with persistent inflammation. They also noted possible complications such as devitalisation of the retained tooth section, ankylosis, and external or replacement resorption. In the present case, the palatal position of the supernumerary element provided a favorable surgical approach: the fusion line was accessible from the palatal aspect, allowing sectioning without compromising the labial periodontal attachment of the lateral incisor. The preservation of the biological width was prioritized during flap repositioning, and the crown restoration with light‐cured glass ionomer cement provided a smooth, well‐contoured surface. Orthodontic space redistribution further facilitated healing by allowing the lateral incisor to move into proper arch alignment, which may have contributed to the favorable soft tissue outcome. However, the potential for late‐onset external root resorption remains a concern, and the patient will require long‐term radiographic monitoring.

The primary goals in managing these anomalies are threefold: restoration of function, achievement of esthetic harmony, and prevention of long‐term complications such as caries, periodontal disease, and pulpal pathology in associated grooves. In this regard, the patient's chief complaint—esthetic disharmony of the anterior maxilla—was successfully resolved through the phased integration of endodontic, surgical, and orthodontic therapies. The preservation of the lateral incisor's periodontal attachment and biological width during surgical sectioning ensured optimal gingival contour, while the subsequent orthodontic intervention facilitated the gradual repositioning of the treated dental unit into its proper arch alignment, restoring both dentoalveolar harmony and patient confidence. While patient satisfaction was assessed through clinical interview and verbal feedback rather than a validated questionnaire, instruments such as the Dental Satisfaction Questionnaire (DSQ) and the SERVQUAL‐based dental satisfaction tool validated in Persian‐speaking populations offer structured approaches for future studies [20].

The prognosis of teeth managed with MTA apical barriers in young patients is favorable, though long‐term data beyond 2 years remain limited. Moore et al. [15] evaluated 22 nonvital immature permanent incisors in 21 children (mean age: 10 years) treated with MTA apical barriers and reported a 95.5% clinical and radiographic success rate over a mean follow‐up of 23.4 months, with significant periapical healing. In a randomized controlled trial, Sun et al. (2024) compared iRoot BP Plus versus MTA apical barriers in 122 young permanent teeth with open apices and chronic apical periodontitis, reporting 12‐month success rates of 98.36% for iRoot BP Plus and 88.52% for MTA [21]. These studies indicate predictable outcomes with MTA apical barriers in immature permanent teeth, with success rates of approximately 88%–97% within 1–2 years. Prognosis is influenced by anatomical factors; Moore et al. [15] identified a significant relationship between nondivergent apical anatomy and ideal MTA plug positioning. In the present case, the presence of separate apical constrictions facilitated controlled MTA placement in each canal, suggesting a favorable long‐term prognosis. Nevertheless, continued follow‐up into adulthood is warranted, as longitudinal data beyond 2 years remain sparse.

A key strength of this report is its demonstration of successful collaborative care between endodontics, oral surgery, and orthodontics, highlighting the value of a team‐based approach in managing rare developmental aberrations. Furthermore, the use of CBCT for preoperative planning and the application of MTA as an apical barrier in teeth with thin dentinal walls represent contemporary evidence‐based practices that enhance treatment predictability.

A limitation of this report is the absence of detailed longitudinal psychosocial data, which could further elucidate the impact of such interventions on patient self‐esteem and quality of life. Nevertheless, follow‐up assessments have consistently revealed stable periodontal and periapical health, with complete soft tissue healing and no evidence of recurrence or pathology. The patient and parents have expressed high satisfaction with the esthetic and functional outcome, reinforcing the clinical efficacy of the multidisciplinary approach.

## Author Contributions

**Mehrfam Khoshkhounejad:** investigation, methodology, writing – original draft, writing – review and editing. **Maryam Babaahmadi:** writing – original draft, methodology. **Somayeh Majidi:** investigation, supervision.

## Funding

The authors have nothing to report.

## Ethics Statement

For clinical cases, the local ethics committee considers that the patient's consent is sufficient.

## Consent

Written informed consent was obtained from the patient to publish this report in accordance with the journal's patient consent policy.

## Conflicts of Interest

The authors declare no conflicts of interest.

## Data Availability

The data that support the findings of this study are available on request from the corresponding author. The data are not publicly available due to privacy or ethical restrictions.
